# 14-Bromo-12-chloro-2,16-dioxa­penta­cyclo­[7.7.5.0^1,21^.0^3,8^.0^10,15^]henicosa-3(8),10,12,14-tetra­ene-7,20-dione

**DOI:** 10.1107/S1600536813010374

**Published:** 2013-04-20

**Authors:** Alan R. Kennedy, Mehmet Akkurt, Shaaban K. Mohamed, Antar A. Abdelhamid, Adel A. E. Marzouk

**Affiliations:** a Department of Pure & Applied Chemistry, University of Strathclyde, 295 Cathedral Street, Glasgow G1 1XL, Scotland; b Department of Physics, Faculty of Sciences, Erciyes University, 38039 Kayseri, Turkey; c Chemistry and Environmental Division, Manchester Metropolitan University, Manchester M1 5GD, England; d Chemistry Department, Faculty of Science, Mini University, 61519 El-Minia, Egypt; e Pharmaceutical Chemistry Department, Faculty of Pharmacy, Al Azhar University, Egypt

## Abstract

In the title compound, C_19_H_16_BrClO_4_, both the fused xanthene rings and one of the cyclo­hexane rings adopt envelope conformations, while the other cyclo­hexane ring is in a chair conformation. In the crystal, mol­ecules are linked by C—H⋯O hydrogen bonds, forming infinite chains running along [10-1] incorporating *R*_2_^2^(16) ring motifs. In addition, C—H⋯π inter­actions and weak π–π stacking inter­actions [centroid–centroid distance = 3.768 (3) Å] help to consolidate the packing.

## Related literature

For similar structures, see: Mohamed *et al.* (2012*b* ▶); Lu *et al.* (2011 ▶); Abdelhamid *et al.* (2011 ▶). For the bioactiviy of xanthenones, see: Mohamed *et al.* (2012*a* ▶); Gobbi *et al.* (2006 ▶); Na (2009 ▶). For ring conformations, see: Cremer and Pople (1975 ▶).
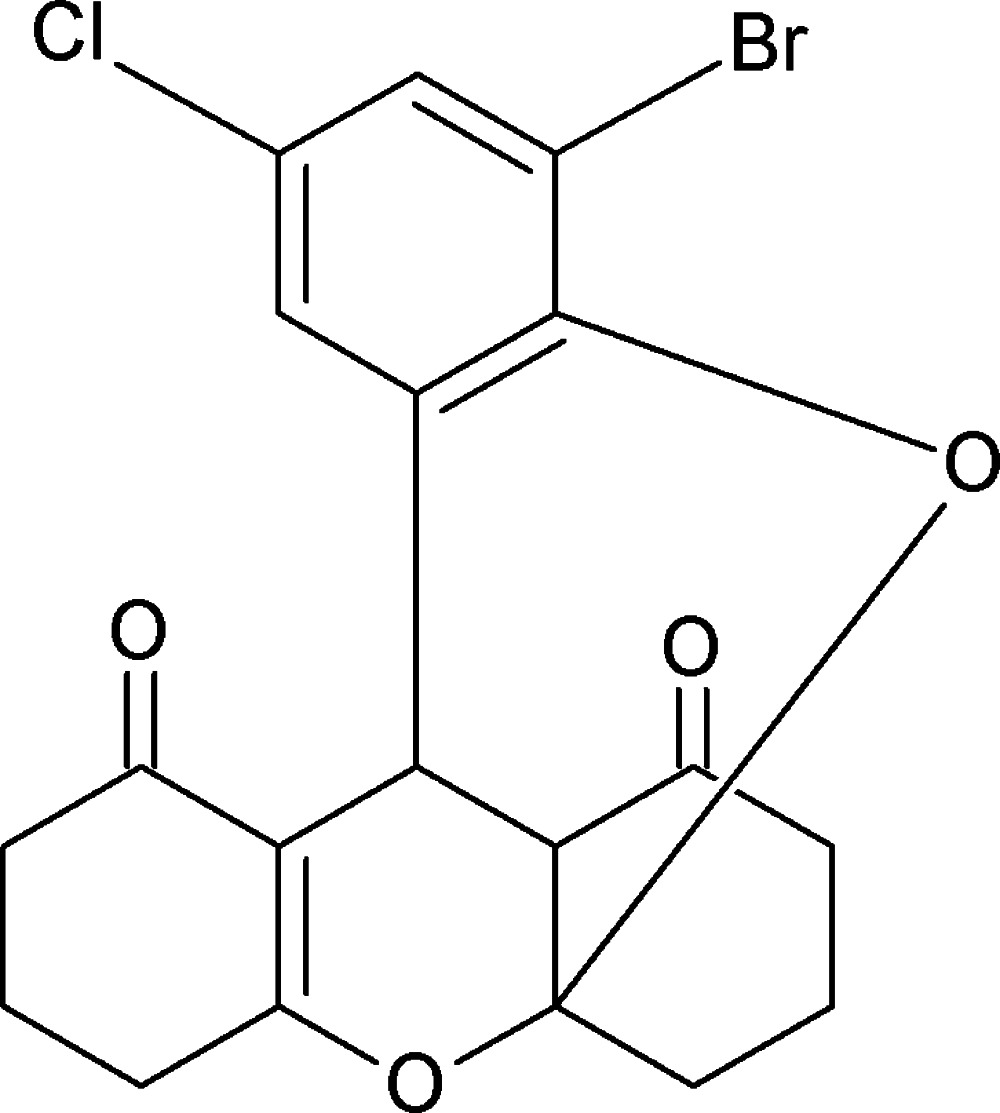


## Experimental

### 

#### Crystal data

C_19_H_16_BrClO_4_
*M* *_r_* = 423.67
Monoclinic, 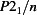

*a* = 10.2741 (6) Å
*b* = 10.2800 (6) Å
*c* = 15.8581 (8) Å
β = 102.073 (5)°
*V* = 1637.85 (16) Å^3^
*Z* = 4
Mo *K*α radiation
μ = 2.70 mm^−1^
*T* = 123 K
0.25 × 0.20 × 0.18 mm

#### Data collection

Oxford Diffraction Xcalibur Eos diffractometer
Absorption correction: multi-scan (*CrysAlis PRO*; Oxford Diffraction, 2010 ▶) *T*_min_ = 0.529, *T*_max_ = 0.616
7243 measured reflections
3516 independent reflections
2547 reflections with *I* > 2σ(*I*)
*R*_int_ = 0.042

#### Refinement

*R*[*F* ^2^ > 2σ(*F* ^2^)] = 0.058
*wR*(*F* ^2^) = 0.134
*S* = 1.04
3516 reflections
226 parameters
H-atom parameters constrained
Δρ_max_ = 0.79 e Å^−3^
Δρ_min_ = −0.73 e Å^−3^

### 

Data collection: *CrysAlis PRO* (Oxford Diffraction, 2010 ▶); cell refinement: *CrysAlis PRO*; data reduction: *CrysAlis PRO*; program(s) used to solve structure: *SHELXS97* (Sheldrick, 2008 ▶); program(s) used to refine structure: *SHELXL97* (Sheldrick, 2008 ▶); molecular graphics: *ORTEP-3 for Windows* (Farrugia, 2012 ▶); software used to prepare material for publication: *WinGX* (Farrugia, 2012 ▶) and *PLATON* (Spek, 2009 ▶).

## Supplementary Material

Crystal structure: contains datablock(s) global, I. DOI: 10.1107/S1600536813010374/hb7072sup1.cif

Structure factors: contains datablock(s) I. DOI: 10.1107/S1600536813010374/hb7072Isup2.hkl

Supplementary material file. DOI: 10.1107/S1600536813010374/hb7072Isup3.cml

Additional supplementary materials:  crystallographic information; 3D view; checkCIF report

## Figures and Tables

**Table 1:** Hydrogen-bond geometry (Å, °) *Cg*3 is the centroid of the C1–C6 benzene ring.

*D*—H⋯*A*	*D*—H	H⋯*A*	*D*⋯*A*	*D*—H⋯*A*
C8—H8*B*⋯O3^i^	0.99	2.53	3.407 (6)	147
C9—H9*B*⋯*Cg*3^ii^	0.99	2.89	3.731 (5)	143

Symmetry codes: (i) 
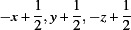
; (ii) 
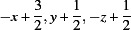
.

**Fig. 1.**
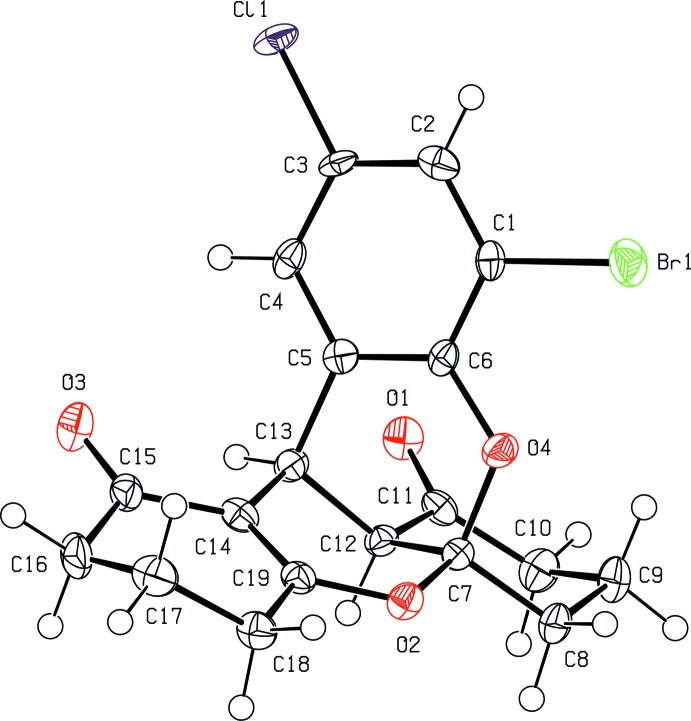
The title compound with displacement ellipsoids for non-H atoms drawn at the 50% probability level.

**Fig. 2.**
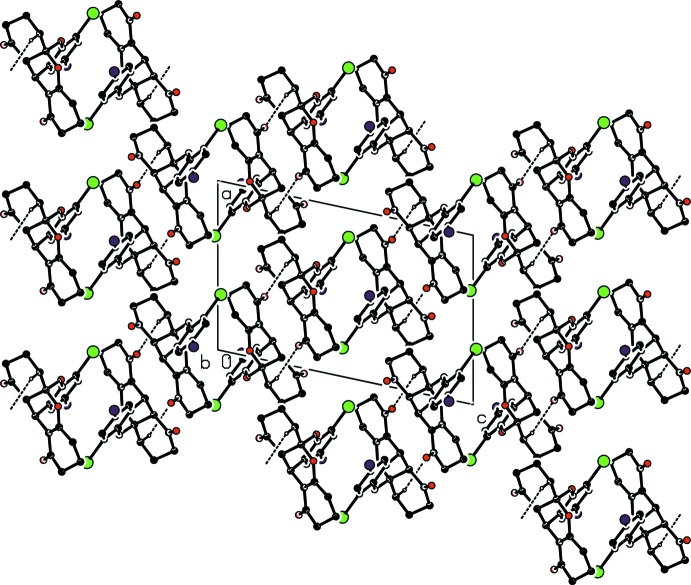
View of the packing and hydrogen bonding diagram of the title compound along the b axis.

## supplementary crystallographic information

### Comment

Xanthenones have very diverse biological profiles, including antihypertensive, anti-oxidative, antithrombotic and anticancer activity, depending on their diverse structures, which are modified by substituents on the ring system (Gobbi *et al.*, 2006; Na, 2009). Following to our earlier study on synthesis of series of the bioactive oxanthenediones (Abdelhamid *et al.*, 2011), acridinediones (Mohamed *et al.*, 2012*a*) and benzopyranes (Mohamed *et al.*, 2012*b*) we became interested in synthesizing the title compound to investigate the relationship between antibacterial activity and structure.

In the title compound, (Fig. 1), the two fused xanthene rings (O2/C7/C12–C14/C19 and O4/C5–C7/C12/C13) adopt envelope conformations [the puckering parameters (Cremer & Pople, 1975) are Q_T_ = 0.522 (5) Å, θ = 127.4 (5) °, φ = 299.1 (6) ° and Q_T_ = 0.539 (5) Å, θ = 125.9 (5) °, φ = 51.2 (6) °, respectively], one (C14–C19) of the cyclohexane rings is also in an envelope conformation with puckering parameters of Q_T_ = 0.440 (5) Å, θ = 129.9 (7) °, φ = 344.4 (9) °, and the other (C7–C12) is in a chair conformation with puckering parameters of Q_T_ = 0.518 (5) Å, θ = 8.0 (6) °, φ = 84 (4). All the bond lengths and bond angles of the title compound are within the expected values and are comparable with those reported for similar structures (Mohamed *et al.*, 2012*b*; Lu *et al.*, 2011; Abdelhamid *et al.*, 2011).

In the crystal structure, long-range C—H···O hydrogen bonds (Table 1, Fig. 2) connect the adjacent molecules into infinite chains running along [101] with *R*^2^_2_(16) ring motifs. C–H···π interactions and weak π-π stacking interactions [*Cg*3···*Cg*3^i^= 3.768 (3) Å; *Cg*3 is a centroid of the C1–C6 benzene ring and symmetry code: (i) = 1 - *x*, 1 - *y*, 1 - *z*] also contribute to the consolidation of the crystal packing.

### Experimental

A mixture of 1 mmol (236 mg) 3-bromo-5-chloro-2-hydroxybenzaldehyde, 1 mmol (112 mg) cyclohexane-1,3-dione and 1 mmol (123 mg) (4-aminophenyl)methanol in 50 ml e thanol was refluxed at 350 K. The reaction progress was monitored by TLC till completion after 5 h. Excess solvent was evaporated under vacuum and the resulted solid was filtered, washed with cold ethanol and recrystallized from ethanol to afford 61% of the title compound. Colourless blocks were obtained by slow evaporation of ethanol solution of (I) at room temperature for two days. M.P. 504 K.

### Refinement

H atoms bound to C atoms were placed at calculated positions [0.95 (aromatic CH), 0.99 (methylene CH_2_) and 1.00 Å (methine CH)] and refined in riding modes with *U*_iso_(H) = 1.2*U*_eq_(C).

### Crystal data

**Table:**

C_19_H_16_BrClO_4_	*F*(000) = 856
*M**_r_* = 423.67	*D*_x_ = 1.718 Mg m^−^^3^
Monoclinic, *P*2_1_/*n*	Mo *K*α radiation, λ = 0.71073 Å
Hall symbol: -P 2yn	Cell parameters from 2011 reflections
*a* = 10.2741 (6) Å	θ = 3.0–28.8°
*b* = 10.2800 (6) Å	µ = 2.70 mm^−^^1^
*c* = 15.8581 (8) Å	*T* = 123 K
β = 102.073 (5)°	Block, colourless
*V* = 1637.85 (16) Å^3^	0.25 × 0.20 × 0.18 mm
*Z* = 4	

### Data collection

**Table:**

Oxford Diffraction Xcalibur Eos diffractometer	3516 independent reflections
Radiation source: Enhance (Mo) X-ray Source	2547 reflections with *I* > 2σ(*I*)
Graphite monochromator	*R*_int_ = 0.042
Detector resolution: 16.0727 pixels mm^-1^	θ_max_ = 27.0°, θ_min_ = 3.3°
ω scans	*h* = −13→13
Absorption correction: multi-scan (*CrysAlis PRO*; Oxford Diffraction, 2010)	*k* = −11→12
*T*_min_ = 0.529, *T*_max_ = 0.616	*l* = −19→20
7243 measured reflections	

### Refinement

**Table:**

Refinement on *F*^2^	Primary atom site location: structure-invariant direct methods
Least-squares matrix: full	Secondary atom site location: difference Fourier map
*R*[*F*^2^ > 2σ(*F*^2^)] = 0.058	Hydrogen site location: inferred from neighbouring sites
*wR*(*F*^2^) = 0.134	H-atom parameters constrained
*S* = 1.04	*w* = 1/[σ^2^(*F*_o_^2^) + (0.0456*P*)^2^ + 3.8105*P*] where *P* = (*F*_o_^2^ + 2*F*_c_^2^)/3
3516 reflections	(Δ/σ)_max_ < 0.001
226 parameters	Δρ_max_ = 0.79 e Å^−^^3^
0 restraints	Δρ_min_ = −0.73 e Å^−^^3^

### Special details

**Table:**

Geometry. Bond distances, angles *etc*. have been calculated using the rounded fractional coordinates. All su's are estimated from the variances of the (full) variance-covariance matrix. The cell e.s.d.'s are taken into account in the estimation of distances, angles and torsion angles
Refinement. Refinement on *F*^2^ for ALL reflections except those flagged by the user for potential systematic errors. Weighted *R*-factors *wR* and all goodnesses of fit *S* are based on *F*^2^, conventional *R*-factors *R* are based on *F*, with *F* set to zero for negative *F*^2^. The observed criterion of *F*^2^ > σ(*F*^2^) is used only for calculating -*R*-factor-obs *etc*. and is not relevant to the choice of reflections for refinement. *R*-factors based on *F*^2^ are statistically about twice as large as those based on *F*, and *R*-factors based on ALL data will be even larger.

### Fractional atomic coordinates and isotropic or equivalent isotropic displacement parameters (Å^2^)

**Table:**

	*x*	*y*	*z*	*U*_iso_*/*U*_eq_	
Br1	0.83070 (5)	0.65176 (6)	0.51190 (3)	0.0317 (2)	
Cl1	0.48771 (12)	0.24296 (11)	0.40547 (8)	0.0239 (4)	
O1	0.5310 (3)	0.6514 (3)	0.1626 (2)	0.0248 (11)	
O2	0.4643 (3)	0.9536 (3)	0.37446 (19)	0.0204 (10)	
O3	0.1281 (3)	0.6340 (4)	0.3173 (2)	0.0309 (12)	
O4	0.6260 (3)	0.7948 (3)	0.3862 (2)	0.0222 (10)	
C1	0.6710 (4)	0.5856 (5)	0.4453 (3)	0.0192 (14)	
C2	0.6398 (5)	0.4564 (5)	0.4527 (3)	0.0209 (16)	
C3	0.5230 (5)	0.4094 (4)	0.4018 (3)	0.0180 (14)	
C4	0.4377 (5)	0.4900 (5)	0.3470 (3)	0.0199 (16)	
C5	0.4683 (5)	0.6205 (4)	0.3418 (3)	0.0174 (14)	
C6	0.5875 (4)	0.6689 (4)	0.3903 (3)	0.0165 (14)	
C7	0.5465 (4)	0.8852 (5)	0.3268 (3)	0.0186 (14)	
C8	0.6430 (5)	0.9822 (5)	0.3029 (3)	0.0224 (16)	
C9	0.7320 (5)	0.9167 (5)	0.2502 (3)	0.0258 (17)	
C10	0.6512 (5)	0.8501 (5)	0.1700 (3)	0.0273 (17)	
C11	0.5501 (5)	0.7597 (5)	0.1918 (3)	0.0188 (14)	
C12	0.4649 (4)	0.8147 (4)	0.2507 (3)	0.0173 (12)	
C13	0.3782 (4)	0.7170 (5)	0.2854 (3)	0.0189 (14)	
C14	0.2961 (4)	0.7914 (5)	0.3381 (3)	0.0185 (14)	
C15	0.1663 (5)	0.7408 (5)	0.3456 (3)	0.0218 (14)	
C16	0.0825 (5)	0.8278 (5)	0.3889 (3)	0.0297 (17)	
C17	0.1648 (5)	0.9056 (5)	0.4622 (3)	0.0293 (17)	
C18	0.2719 (5)	0.9826 (5)	0.4319 (3)	0.0226 (16)	
C19	0.3432 (5)	0.9016 (5)	0.3786 (3)	0.0185 (14)	
H2	0.69700	0.40090	0.49180	0.0250*	
H4	0.35780	0.45610	0.31280	0.0240*	
H8A	0.69840	1.01970	0.35590	0.0270*	
H8B	0.59310	1.05390	0.26900	0.0270*	
H9A	0.78860	0.85130	0.28640	0.0310*	
H9B	0.79120	0.98260	0.23250	0.0310*	
H10A	0.60600	0.91700	0.12930	0.0320*	
H10B	0.71220	0.80100	0.14090	0.0320*	
H12	0.40380	0.88020	0.21640	0.0210*	
H13	0.31860	0.67080	0.23660	0.0220*	
H16A	0.01760	0.77390	0.41160	0.0360*	
H16B	0.03170	0.88840	0.34570	0.0360*	
H17A	0.10610	0.96580	0.48570	0.0350*	
H17B	0.20660	0.84580	0.50900	0.0350*	
H18A	0.23100	1.05820	0.39760	0.0270*	
H18B	0.33640	1.01600	0.48260	0.0270*	

### Atomic displacement parameters (Å^2^)

**Table:**

	*U*^11^	*U*^22^	*U*^33^	*U*^12^	*U*^13^	*U*^23^
Br1	0.0228 (3)	0.0413 (4)	0.0276 (3)	−0.0031 (2)	−0.0026 (2)	0.0050 (3)
Cl1	0.0344 (7)	0.0103 (6)	0.0300 (6)	−0.0019 (5)	0.0134 (5)	−0.0010 (5)
O1	0.0262 (19)	0.026 (2)	0.0228 (17)	−0.0010 (16)	0.0067 (14)	−0.0059 (16)
O2	0.0222 (18)	0.0182 (18)	0.0219 (17)	−0.0016 (14)	0.0075 (14)	−0.0044 (14)
O3	0.024 (2)	0.034 (2)	0.036 (2)	−0.0078 (17)	0.0092 (16)	−0.0022 (18)
O4	0.0228 (18)	0.0182 (18)	0.0230 (17)	−0.0041 (14)	−0.0012 (14)	0.0016 (14)
C1	0.014 (2)	0.025 (3)	0.020 (2)	−0.002 (2)	0.0066 (19)	−0.001 (2)
C2	0.024 (3)	0.024 (3)	0.018 (2)	0.009 (2)	0.012 (2)	0.004 (2)
C3	0.026 (3)	0.008 (2)	0.023 (2)	0.0001 (19)	0.012 (2)	0.0006 (19)
C4	0.020 (3)	0.020 (3)	0.023 (2)	−0.005 (2)	0.012 (2)	−0.005 (2)
C5	0.020 (2)	0.019 (3)	0.016 (2)	0.0013 (19)	0.0102 (19)	−0.0002 (19)
C6	0.016 (2)	0.018 (3)	0.018 (2)	−0.0022 (19)	0.0096 (18)	−0.003 (2)
C7	0.017 (2)	0.016 (3)	0.023 (2)	−0.0007 (19)	0.005 (2)	0.001 (2)
C8	0.020 (3)	0.022 (3)	0.024 (2)	−0.006 (2)	0.002 (2)	−0.004 (2)
C9	0.019 (3)	0.031 (3)	0.029 (3)	−0.005 (2)	0.009 (2)	−0.002 (2)
C10	0.028 (3)	0.028 (3)	0.026 (3)	−0.004 (2)	0.006 (2)	−0.002 (2)
C11	0.018 (2)	0.023 (3)	0.014 (2)	0.004 (2)	0.0001 (18)	0.000 (2)
C12	0.019 (2)	0.016 (2)	0.017 (2)	−0.0011 (19)	0.0037 (18)	0.0021 (19)
C13	0.020 (2)	0.017 (3)	0.019 (2)	−0.002 (2)	0.0026 (19)	0.0011 (19)
C14	0.020 (2)	0.023 (3)	0.013 (2)	0.005 (2)	0.0043 (18)	0.003 (2)
C15	0.017 (2)	0.027 (3)	0.020 (2)	−0.002 (2)	0.0009 (19)	0.007 (2)
C16	0.015 (3)	0.039 (3)	0.037 (3)	0.000 (2)	0.010 (2)	0.008 (3)
C17	0.031 (3)	0.032 (3)	0.028 (3)	0.007 (2)	0.013 (2)	0.006 (2)
C18	0.024 (3)	0.024 (3)	0.022 (2)	0.004 (2)	0.010 (2)	0.000 (2)
C19	0.018 (2)	0.021 (3)	0.017 (2)	0.003 (2)	0.0045 (19)	0.005 (2)

### Geometric parameters (Å, º)

**Table:**

Br1—C1	1.883 (5)	C14—C15	1.459 (7)
Cl1—C3	1.753 (4)	C14—C19	1.342 (7)
O1—C11	1.206 (6)	C15—C16	1.503 (7)
O2—C7	1.430 (6)	C16—C17	1.515 (7)
O2—C19	1.368 (6)	C17—C18	1.513 (7)
O3—C15	1.220 (6)	C18—C19	1.484 (7)
O4—C6	1.359 (5)	C2—H2	0.9500
O4—C7	1.448 (6)	C4—H4	0.9500
C1—C2	1.377 (7)	C8—H8A	0.9900
C1—C6	1.385 (6)	C8—H8B	0.9900
C2—C3	1.386 (7)	C9—H9A	0.9900
C3—C4	1.375 (7)	C9—H9B	0.9900
C4—C5	1.384 (7)	C10—H10A	0.9900
C5—C6	1.395 (7)	C10—H10B	0.9900
C5—C13	1.515 (7)	C12—H12	1.0000
C7—C8	1.509 (7)	C13—H13	1.0000
C7—C12	1.504 (6)	C16—H16A	0.9900
C8—C9	1.519 (7)	C16—H16B	0.9900
C9—C10	1.528 (7)	C17—H17A	0.9900
C10—C11	1.487 (7)	C17—H17B	0.9900
C11—C12	1.517 (7)	C18—H18A	0.9900
C12—C13	1.520 (6)	C18—H18B	0.9900
C13—C14	1.513 (7)		
			
C7—O2—C19	118.5 (4)	O2—C19—C14	123.2 (4)
C6—O4—C7	120.8 (3)	O2—C19—C18	111.7 (4)
Br1—C1—C2	119.5 (4)	C14—C19—C18	125.1 (5)
Br1—C1—C6	118.8 (4)	C1—C2—H2	121.00
C2—C1—C6	121.7 (4)	C3—C2—H2	121.00
C1—C2—C3	118.4 (4)	C3—C4—H4	120.00
Cl1—C3—C2	118.8 (4)	C5—C4—H4	120.00
Cl1—C3—C4	120.0 (4)	C7—C8—H8A	110.00
C2—C3—C4	121.2 (4)	C7—C8—H8B	110.00
C3—C4—C5	119.9 (5)	C9—C8—H8A	110.00
C4—C5—C6	119.8 (4)	C9—C8—H8B	110.00
C4—C5—C13	123.5 (4)	H8A—C8—H8B	108.00
C6—C5—C13	116.7 (4)	C8—C9—H9A	109.00
O4—C6—C1	118.1 (4)	C8—C9—H9B	109.00
O4—C6—C5	123.0 (4)	C10—C9—H9A	109.00
C1—C6—C5	118.9 (4)	C10—C9—H9B	109.00
O2—C7—O4	106.7 (3)	H9A—C9—H9B	108.00
O2—C7—C8	107.4 (4)	C9—C10—H10A	109.00
O2—C7—C12	111.7 (3)	C9—C10—H10B	109.00
O4—C7—C8	106.1 (3)	C11—C10—H10A	109.00
O4—C7—C12	110.9 (4)	C11—C10—H10B	109.00
C8—C7—C12	113.7 (4)	H10A—C10—H10B	108.00
C7—C8—C9	110.4 (4)	C7—C12—H12	107.00
C8—C9—C10	111.8 (4)	C11—C12—H12	107.00
C9—C10—C11	111.8 (4)	C13—C12—H12	107.00
O1—C11—C10	123.6 (5)	C5—C13—H13	110.00
O1—C11—C12	120.8 (4)	C12—C13—H13	110.00
C10—C11—C12	115.6 (4)	C14—C13—H13	110.00
C7—C12—C11	112.2 (4)	C15—C16—H16A	109.00
C7—C12—C13	107.4 (4)	C15—C16—H16B	109.00
C11—C12—C13	115.7 (4)	C17—C16—H16A	109.00
C5—C13—C12	108.3 (4)	C17—C16—H16B	109.00
C5—C13—C14	110.3 (4)	H16A—C16—H16B	108.00
C12—C13—C14	107.6 (4)	C16—C17—H17A	109.00
C13—C14—C15	119.3 (4)	C16—C17—H17B	109.00
C13—C14—C19	120.3 (4)	C18—C17—H17A	109.00
C15—C14—C19	120.4 (4)	C18—C17—H17B	109.00
O3—C15—C14	121.3 (5)	H17A—C17—H17B	108.00
O3—C15—C16	122.2 (5)	C17—C18—H18A	109.00
C14—C15—C16	116.5 (4)	C17—C18—H18B	109.00
C15—C16—C17	112.6 (4)	C19—C18—H18A	109.00
C16—C17—C18	111.0 (4)	C19—C18—H18B	109.00
C17—C18—C19	111.5 (4)	H18A—C18—H18B	108.00
			
C19—O2—C7—O4	−88.4 (4)	O4—C7—C12—C11	−70.2 (5)
C19—O2—C7—C8	158.3 (4)	O4—C7—C12—C13	58.0 (4)
C19—O2—C7—C12	32.9 (5)	C8—C7—C12—C11	49.2 (5)
C7—O2—C19—C14	−2.0 (7)	C8—C7—C12—C13	177.4 (4)
C7—O2—C19—C18	178.7 (4)	C7—C8—C9—C10	55.9 (5)
C7—O4—C6—C1	176.9 (4)	C8—C9—C10—C11	−53.0 (6)
C7—O4—C6—C5	−3.2 (6)	C9—C10—C11—O1	−134.8 (5)
C6—O4—C7—O2	96.0 (4)	C9—C10—C11—C12	48.4 (6)
C6—O4—C7—C8	−149.7 (4)	O1—C11—C12—C7	136.7 (5)
C6—O4—C7—C12	−25.8 (5)	O1—C11—C12—C13	13.0 (6)
Br1—C1—C2—C3	−178.8 (4)	C10—C11—C12—C7	−46.4 (5)
C6—C1—C2—C3	1.6 (7)	C10—C11—C12—C13	−170.0 (4)
Br1—C1—C6—O4	0.9 (6)	C7—C12—C13—C5	−62.3 (5)
Br1—C1—C6—C5	−179.1 (3)	C7—C12—C13—C14	57.0 (4)
C2—C1—C6—O4	−179.5 (4)	C11—C12—C13—C5	63.8 (5)
C2—C1—C6—C5	0.5 (7)	C11—C12—C13—C14	−176.9 (4)
C1—C2—C3—Cl1	175.9 (4)	C5—C13—C14—C15	−90.3 (5)
C1—C2—C3—C4	−2.0 (7)	C5—C13—C14—C19	88.6 (6)
Cl1—C3—C4—C5	−177.6 (4)	C12—C13—C14—C15	151.7 (4)
C2—C3—C4—C5	0.3 (8)	C12—C13—C14—C19	−29.4 (6)
C3—C4—C5—C6	1.9 (7)	C13—C14—C15—O3	7.1 (7)
C3—C4—C5—C13	−178.0 (4)	C13—C14—C15—C16	−172.2 (4)
C4—C5—C6—O4	177.7 (4)	C19—C14—C15—O3	−171.9 (5)
C4—C5—C6—C1	−2.3 (7)	C19—C14—C15—C16	8.8 (7)
C13—C5—C6—O4	−2.4 (7)	C13—C14—C19—O2	0.7 (7)
C13—C5—C6—C1	177.6 (4)	C13—C14—C19—C18	180.0 (4)
C4—C5—C13—C12	−144.7 (5)	C15—C14—C19—O2	179.7 (4)
C4—C5—C13—C14	97.8 (5)	C15—C14—C19—C18	−1.1 (8)
C6—C5—C13—C12	35.4 (6)	O3—C15—C16—C17	144.8 (5)
C6—C5—C13—C14	−82.2 (5)	C14—C15—C16—C17	−35.9 (6)
O2—C7—C8—C9	−178.8 (4)	C15—C16—C17—C18	54.9 (6)
O4—C7—C8—C9	67.5 (5)	C16—C17—C18—C19	−46.2 (6)
C12—C7—C8—C9	−54.7 (5)	C17—C18—C19—O2	−160.1 (4)
O2—C7—C12—C11	171.0 (4)	C17—C18—C19—C14	20.5 (7)
O2—C7—C12—C13	−60.8 (5)		

### Hydrogen-bond geometry (Å, º)

Cg3 is the centroid of the C1–C6 benzene ring.

**Table:**

*D*—H···*A*	*D*—H	H···*A*	*D*···*A*	*D*—H···*A*
C8—H8*B*···O3^i^	0.99	2.53	3.407 (6)	147
C9—H9*B*···*Cg*3^ii^	0.99	2.89	3.731 (5)	143

Symmetry codes: (i) −*x*+1/2, *y*+1/2, −*z*+1/2; (ii) −*x*+3/2, *y*+1/2, −*z*+1/2.
